# Mammography Image-Based Diagnosis of Breast Cancer Using Machine Learning: A Pilot Study

**DOI:** 10.3390/s22010203

**Published:** 2021-12-28

**Authors:** Maha M. Alshammari, Afnan Almuhanna, Jamal Alhiyafi

**Affiliations:** 1 Computational Unit, Department of Environmental Health, Institute for Research and Medical Consultations, Imam Abdulrahman Bin Faisal University, Dammam 31441, Saudi Arabia; 2 Department of Radiology, College of Medicine, Imam Abdulrahman Bin Faisal University, Dammam 31441, Saudi Arabia; amuhanna@iau.edu.sa; 3 Department of Computer Science, Kettering University, Flint, MI 48504, USA; jalhiyafi@kettering.edu

**Keywords:** breast cancer, machine learning, classification, support vector machine, decision tree, K-nearest neighbor, Naïve Bayes, discriminant analysis, benign, malignant

## Abstract

A tumor is an abnormal tissue classified as either benign or malignant. A breast tumor is one of the most common tumors in women. Radiologists use mammograms to identify a breast tumor and classify it, which is a time-consuming process and prone to error due to the complexity of the tumor. In this study, we applied machine learning-based techniques to assist the radiologist in reading mammogram images and classifying the tumor in a very reasonable time interval. We extracted several features from the region of interest in the mammogram, which the radiologist manually annotated. These features are incorporated into a classification engine to train and build the proposed structure classification models. We used a dataset that was not previously seen in the model to evaluate the accuracy of the proposed system following the standard model evaluation schemes. Accordingly, this study found that various factors could affect the performance, which we avoided after experimenting all the possible ways. This study finally recommends using the optimized Support Vector Machine or Naïve Bayes, which produced 100% accuracy after integrating the feature selection and hyper-parameter optimization schemes.

## 1. Introduction

Breast cancer was ranked the fifth among the most common causes of cancer death based on World Health Organization (WHO) statistics [1]. Breast cancer is considered a frequent cancer, where 2.1 million women worldwide get affected every year [2]. The estimated mortality rate of this cause in 2020 was 685,000, which was approximately 13.6% of all cancer deaths that involved women [2]. In the Kingdom of Saudi Arabia, it represents about 15.9% of the detected cases of cancer, according to the latest Ministry of Health statistics in 2014 [3]. It is ranked high among various types of cancer that affect Saudi citizens [3]. Therefore, early detection of breast cancer is essential to reduce its mortality rate and increase the recovery rate [2,4]. Most diagnosed women are detected in the late stages due to resource limitations [2]. The screening technique is one of the earliest detection strategies before seeing the breast cancer symptoms. Many tools are used as screening techniques, such as mammography, which is considered the most common tool for early detection of breast cancer [2,4]. Mammography images produce a significant amount of information that a radiologist has to analyze and assess comprehensively in a short time to detect the type of tumor in its early stage, while the multistage process usually takes a longer time.

This paper proposes a machine learning (ML)-based computer-aided diagnosis (CAD) system to assist radiologists in classifying breast tumors as either benign or malignant using mammography data. It can rank the type of tumor without going through multiple stages that may cause treatment delay. To accomplish the aim of this paper, we propose a new method for developing a CAD system for breast cancer. The main idea of this method is to integrate the radiologist in the tumor annotation step in the CAD’s workflow since an efficient CAD system should not completely exclude the radiologist’s role [5]. We extracted several features using the annotated tumor to train the classifier for classification purposes. We tested the performance of five classifiers using several experiments. These classifiers are Decision Tree (DT), K-Nearest Neighbor (KNN), Support Vector Machine (SVM), Naïve Bayes (NB), and Discriminant Analysis (DA).

We selected the classifiers based on reasonable grounds. We chose them to represent the different models’ schemes, such as statistical-based, divide and conquer-based, instance-based learning, and linear classification-based [6].

In addition, we selected each classifier based on its advantages. DT is based on a divide and conquer (rules-based) scheme [6]. It is one of the popular approaches for classification [7], and it is easy to understand due to its mechanism [8]. Learning processes based on instance are represented by the KNN technique [6], which is a more straightforward technique that identifies the group’s membership using similarity [9,10], especially with the numeric values [6]. NB is a probabilistic-based scheme [6], where it works according to a simple approach [10,11] that uses probabilistic theory [6,12]. It works effectively in practice with excellent results for classification [6,13]. It performed well with breast cancer problems in previous studies [10]. Based on the linear classification principles, SVM and DA were selected. Mathematically, SVM has a strong basis in theory. It showed very high performance in various disease diagnoses, image processing, etc. [13]. On the other hand, the discriminant function works well for group separation purposes, making it significant [14].

The proposed ML-based CAD system can assist the radiologist in Saudi Arabia in interpreting such images and thus improve the performance of diagnosing breast cancer and support faster decision-making for the treatment.

We used MATLAB in this work. Three experiments were done on the above classification techniques using different approaches to improve the performance. The process started after extracting 12 features based on the breast density, shape, and texture using a local real mammography dataset that never has been used previously. Each experiment consisted of several tests using different criteria. Accordingly, this paper found that various factors can affect performance. After testing all possible ways, these factors were avoided in the third experiment, clarifying those factors. After integrating the feature selection and hyper-parameter optimization functions, we obtained excellent accuracy for all tested classifiers. We did a proving test using unlabeled samples, and a radiologist reviewed the results and indicated that the optimized SVM and NB obtained 100% accuracy.

The remaining part of this paper is organized as follows: Section 2 reviews several related works; Section 3 explains the proposed method; Section 4 contains the experimental studies and results; Section 5 discusses the findings of the proposed method and the recommended models; and Section 6 concludes this paper.

## 2. Background

CAD systems play an essential role in the preliminary stages of detecting disease. Different techniques of ML can be used individually or in hybrid. The technique selection depends on the available data, its ambiguity, and the ability to select useful features [15,16].

One of the most critical CAD systems is the system that aims to detect or diagnose breast cancer utilizing computer technology to detect any abnormalities in breast tissues [17]. The systems are classified as one of three systems: abnormality-detection systems classify the abnormality to either benign or malignant or detect and classify the class of abnormalities [18]. The CAD system is an essential alternative to the biopsy due to the possible effects of biopsy, such as discomfort to the patient, infection, bleeding, and the time needed to get the results after laboratory analysis [19].

**Figure 1:**
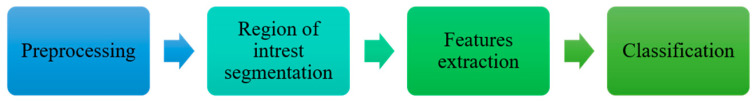
Mammography-based CAD system.

Significant early stage detection of breast cancer relies on the breast imaging methods used. The accuracy of interpretation by radiologists of the mammograms depends on different factors. Mammograms have been included in CAD systems [20], which is one of the most critical advances in CAD systems to serve this purpose [18,20]. The typical stages in mammography-based CAD systems are shown in Figure 1 [18].

Many classification techniques can be examined associated with the extracted features to evaluate the performance of each of them. The most popular classification techniques that we reviewed are the following.

### 2.1. Decision Trees (DT)

A Decision Tree is divided based on the rules [6]. It is a structural method represented as a flowchart similar to the tree and more like the human’s logic, making it easy to understand [8].

The critical characteristic of a Decision Tree is how it is easy to read by people due to its mechanism [8]. It combines categorical and numerical data [6,7]. It has a few issues and challenges, such as classification problems with high dimensionalities and unbalanced data [8].

### 2.2. K-Nearest Neighboring (KNN)

KNN is an instance-based classifier [6]. It is an efficient prediction model and one of the most straightforward techniques for learning artificial algorithms based on similarity [9,10,21]. The most affected factor of KNN, especially in medical applications, is K’s value, and there are different ways of selecting its value. The easiest way could be done by running the KNN algorithm many times and changing the K value every time until the optimal value is found [21].

It performs very well in some cases. It has a few limitations, such as the time it takes to check all instances with each instance classification; also, its sensitivity to any type of attribute, correlated or not, and any mistake in specifying the value of k can affect its performance [9].

### 2.3. Support Vector Machine (SVM)

SVM is statistical learning-based model that works according to the supervised learning approach [13,22,23,24]. It belongs to the linear classification models, which works with low-dimensional and uncomplicated data that can easily separate the data space. However, it can also deal with a non-linear classifications that has high-dimensional and complex data by using a kernel function to transform the data into another high-dimensional data space that can be separated [13,22,23,24,25]. It represents the data items as points and classifies them with their belonging classes in the space. It works by finding the hyperplane that separates them by maximizing the margins that differentiate between the classes [22,23,26,27,28]. 

It is a powerful and robust model; its efficiency has been proven in various applications, such as financial, biological, image processing, bioinformatics, face recognition, text mining, and disease diagnosis for different medical fields, especially in cancer prediction and classification. The SVM efficiency in the applications mentioned above makes it a prevalent technique [13,29,30,31]. Mathematically, it has a strong basis in statistical learning theory [13].

### 2.4. Naïve Bayes (NB)

Naïve Bayes is a simple supervised learning technique for classification based on the probabilistic theorem [6,10,11]. It is a very efficient technique, especially when combined with feature selection functions [6].

It outperforms other powerful techniques due to many reasons, such as being simple to apply, fast, and producing high accuracy. It has been used in many areas, such as disease diagnosis, classification of RNA sequences, and spam filtering [32].

### 2.5. Discriminant Analysis (DA)

Discriminant Analysis is appropriate to be used in supervised learning for data grouping tasks that have known classes. It works by predefined groups using original data to classify new unlabeled instances [33,34]. It is a statistically based technique and one of the linear classifiers [14].

This technique is multivariate; it works by finding the best combinations of the attributes used as predictors to maximize the separation between the given groups of data, which is needed to predict the group membership of any new instance. The best combinations between the given attributes are called discriminant functions [14]. 

## 3. Related Work

Recently, many researchers have highlighted intelligent systems. Many disease diagnoses and decision support have included studies to diagnose tumors, especially breast tumors. Some studies focused on using a single technique, and others used a combination of techniques to achieve the highest reliability of the results depending on different data.

This section reviewed the state-of-the-art studies in breast cancer diagnosis using machine learning techniques from 2007 to the present time. The review process started in 2007 due to the gap in studies done before 2007. In 2007, this subject became very fertile and an interesting research subject. Awareness of early detection of breast cancer increased in the world. There is a need to employ artificial intelligence applications to support this process and help radiologists support their treatment decisions.

In 2007, Polat et al. [35] proposed a hybrid method, where he combined a fuzzy-artificial immune system with KNN algorithms. He classified one of the most common datasets, Wisconsin Diagnostic Breast Cancer (WBCD), to examine the proposed method’s accuracy compared to other studies. The proposed technique achieved 99.14% accuracy, and its accuracy was obtained using one of the cross-validation techniques.

Akay [36] proposed the SVM-based model, and it was combined with feature selection. The model was trained and evaluated using different partitions of WBCD. The SVM model achieved 99.51% accuracy.

Keles et al. [19] developed expert system-based neuro-fuzzy rules for diagnosing breast cancer (Ex-DBC) to avoid using biopsy, and it used an excellent performance learning algorithm in disease diagnosis and classification. The proposed system contained three layers: the input layer, the hidden layer containing nine fuzzy-rules, and the last layer representing the output that specified the class of a particular case.

A new Neuro-Fuzzy System (NFS) [37] has been proposed and its performance compared with two other powerful supervised techniques: Radial Basis Function Neural Network (RBFNN) and Adaptive Neuro-Fuzzy Inference System (ANFIS). The training and testing phases for the RBFNN, ANFIS, and NFS techniques were done using the WBCD dataset. NFS achieved the highest classification accuracy, which was 98.4%.

A digital mammography dataset was used to evaluate the new algorithm [38]. The proposed algorithm was based on Non-Subsampled Contourlet Transform (NSCT) and Super Resolution (SR) combined with an AdaBoost classifier. NSCT and SR improved the image quality after automating the extraction of the region of interest (ROI). An accuracy of 91.43% was achieved.

A novel CAD system has been proposed [39] based on the Extreme Learning Machine (ELM) classifier using the available mammography images set. It included several steps: image preprocessing, ROI segmentation, features extraction, features selection, mass classification, and final assessment of the proposed model. The proposed ELM obtained 96.02% accuracy.

Emina and Abdulhamit [40] proposed a two-stage system. First, they used a genetic algorithm to extract the essential and useful features from a dataset containing 569 cases and 32 features. In the second stage, hybrid and individual data mining techniques were used to propose an accurate result. The Rotation Forest technique obtained the highest accuracy, which was 99.48%.

Hybridized SVM algorithms based on Weighted Area Under the Receiver Operating Characteristic Curve Ensemble (WAUCE) were incorporated to build a new model to increase the accuracy and reduce a variety of breast cancer diagnoses [41]. Two published and one real dataset were used to evaluate the proposed model. The highest accuracy achieved was 97.68% by applying a 10-fold validation technique on the Wisconsin Diagnostic Breast Cancer (WDBC) dataset. 

Using the 569 cases and 32 features of the dataset, Joshi and Mehta [42] examined the diagnosis accuracy of the KNN ML technique in the R environment. It was done by employing the most significant dimensionality reduction techniques: Linear Discriminant Analysis (LDA) and Principal Component Analysis (PCA). LDA achieved a better accuracy (97.06%) than PCA.

A novel intelligent model has been proposed. It is based on the combination of an information gain algorithm for features ranking and a simulated annealing genetic algorithm wrapper for selecting the optimal features [43]. The chosen features inferred the cost-sensitive SVM classifier. The Wisconsin Original Breast Cancer (WBC) and WDBC datasets were used to train and test the proposed model. The optimum accuracy was 95.8%, which was obtained with the WBC dataset.

Both Chaurasia and Pal [44] and Abdar et al. [45] used the WDBC dataset to compare different ML techniques’ accuracy. In Abdar et al.’s study [45], an approach of the nested ensemble was applied by using two layers. It contained two to three classifiers that have been combined using Stacking and Vote (SV); the SV-NB-3-Meta Classifier had the best obtained accuracy (98.07%) with the least model building time compared with the other combined algorithms. Chaurasia and Pal [44] compared six ML techniques, including SVM, NB, KNN, DT, and Random Forest (RF); all of them exceeded 90% accuracy.

## 4. Proposed Method

**Figure 2:**
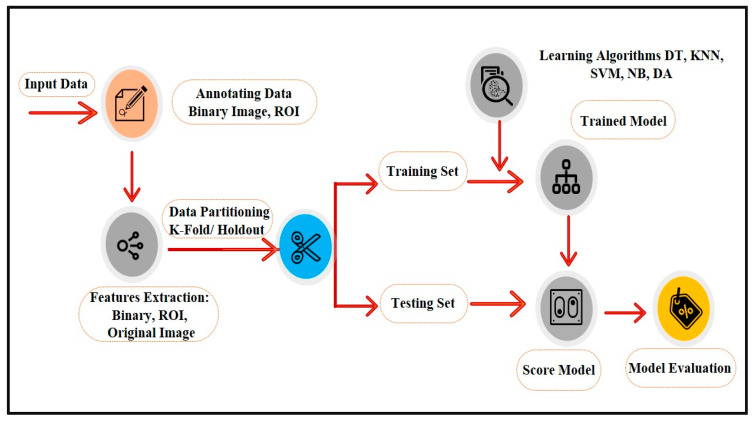
Schematic diagram for the proposed ML-based CAD system.

A series of image processing algorithms were developed to build the proposed ML-based CAD system containing multiple stages. The preliminary stages shown in Figure 2 are discussed in the following subsections.

### 4.1. Annotating Data

One of the reasonable approaches to describe the abnormalities is by human perception. While the radiologists have special training to identify the state of the tissue in the breast from its appearance in a mammogram, many CAD systems used the features described by radiologists to build and train the proposed systems, although they used different terminologies for the same features [46].

Other CAD systems for breast cancer diagnosis based on mammography data used image processing techniques for segmenting and extracting features, which may affect the accuracy due to the tissue ambiguity and complexity.

In this study, the nominated radiologist manually annotated the abnormal region by drawing the border between the tumor and the background of a mammogram.

### 4.2. Features Extraction

In this stage, algorithms were developed to extract several features to characterize the ROI that the radiologist annotated. These features are intensity-based (average intensity, standard deviation, and contrast between the foreground ROI and background), shape-based (degree of circulatory, elongation, length, and diameter), or texture-based features. Texture is a significant property to identify ROI [47,48], and they provide characteristics such as smoothness, coarseness, and regularity of the intensity values in a ROI [26,49]. 

The main problems in texture features analysis are specifying which features are required and the processing type needed for texture recognition [50]. The effective texture features depend on the type of images and effective factors in the research problem. Texture features extraction requires an evaluation of the different features to choose the most effective of them in a particular case [50]. The image processing algorithms that need to be implemented to extract those features will be discussed next.

### 4.3. Classification

Many classification techniques can be examined associated with the extracted features to evaluate the performance of each of them. The classification techniques aforementioned in the background section were chosen to be examined to identify the most optimal solution for this application. The extracted features were used to build and train the proposed classification models.

### 4.4. Cross Validation

The cross-validation technique was used for evaluating the performance validation of a specific model for a sample of data for future data. It was done by dividing the data into groups: the training set, which was used to apply the model, and the testing set, which was used to calculate the results error ratio. This will help improve the proposed model to obtain the highest performance.

## 5. Experiments

MATLAB software was used, including the Image processing toolbox, Graphic toolbox, Mathematic toolbox, and Statistics and ML toolbox to implement the proposed method, including all steps from preprocessing to evaluation and improving the performance. 

In this section, we describe all the experiments that were done. It begins with describing the collected dataset and how it was preprocessed to test the proposed model. Then, we explain how we implemented the experiments and improved them until we obtained the best performance.

### 5.1. Dataset Description

We received ethical approval from the Standing Committee for Research Ethics on Living Creatures (SCRELC) at Imam Abdulrahman Bin Faisal University (IAU) (reference number IRB-PGS-2018-09-292). Then, the mammography dataset used in this study was collected from King Fahd Hospital of the University (KFUH) affiliated to IAU in Khobar, Kingdom of Saudi Arabia. The collected data are new and have not been published before. We collected it from consecutive recent patients who had digital mammograms, considering the variety between the chosen samples in terms of density, shape, and tumor size. The collected dataset contains 42 instances, 21 benign (BIRADS 2) and 21 malignant (BIRADS 5). Each instance has 4 two dimensional images for the right and left breast. So, there were two different views of the images for each side (mediolateral–oblique (MLO) and cranial–caudal (CC)).

Whether the view of the image was MLO or CC, we selected the image that had a clear tumor in every instance to prepare the data for the tumor annotating step.

### 5.2. Data Annotation

**Figure 3:**
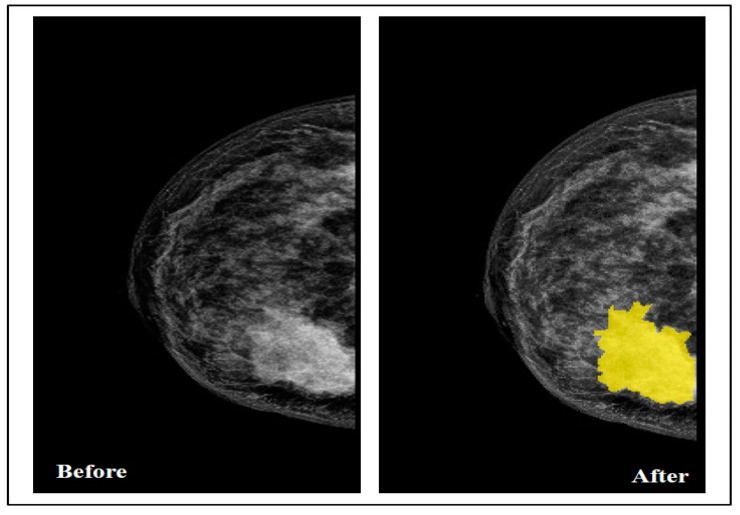
Breast before and after drawing the tumor border.

By collaborating with the radiologist, the Segmenter tool in MATLAB was used in this step to manually annotate the tumor region by drawing its border in all the selected images, as seen in Figure 3.

**Figure 4:**
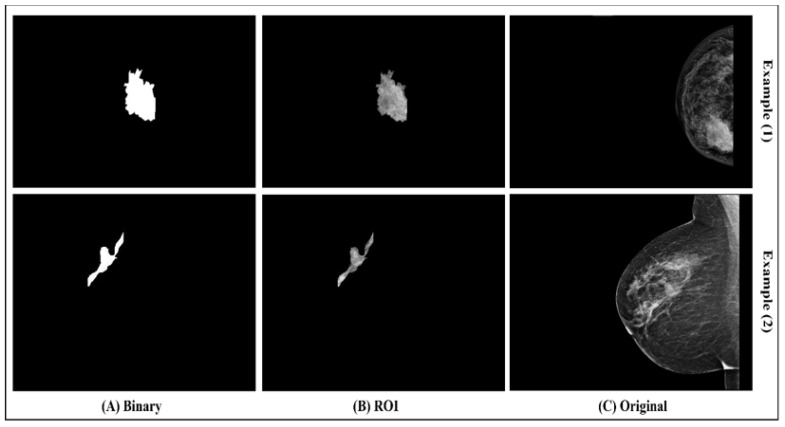
(**A**) Binary image; (**B**) ROI; (**C**) original image.

After drawing the tumor border, the segmented tumor area (ROI) was converted to binary and masked images, as seen in Figure 4. The image conversion was done using the function generator by the Segmenter tool. The masked image represents only the ROI and it was extracted from the breast without its background. The binary and masked images were exported to use in the features extraction step.

### 5.3. Features Extraction

**Figure 5:**
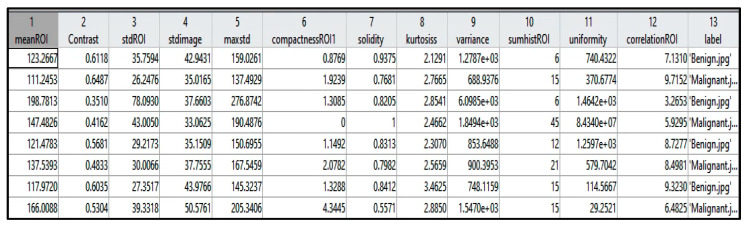
Samples of the extracted features from a few cases.

Table 1 identifies each extracted feature with the factor that describes it, and Figure 5 shows a sample of the extracted values from a few instances. The extracted features were selected based on the tumor density, shape, and texture.

**Table 1:** Extracted features.

Based	Feature	Description	Equation	Equation Number
Density	Mean	The mean is one of the most basic notions in experimental sciences. It is used in many applications of everyday life [51]. It is an average of the pixels’ density value. Equation (1) is used to calculate the mean of ROI density and the mean of the whole image of breast density. Equation (2) is the calculated proportion of dissimilarity between the ROI mean and the image mean.	$\bar{X}=\frac{1}{n}\sum_{i=1}^{n}X_{i}$ X vector contains the value of n pixels where n represents the number of image pixels and $X_{i}$ each pixel density.	(1)
			$Mean\;Contrast=\frac{ROI\;\bar{X}}{image\;\bar{X}}$	(2)
	SD	Standard deviation is used to measure the degree of dispersion and variation of the image [52]. This value represents the mean distance between the pixel and the mean. Equation (3) is the calculated SD (ROI) and SD (image). Equation (4) is the calculated Max (SD ROI).	$SD=\sqrt{\frac{1}{n}\sum_{i=1}^{n}{\left(X_{i}-\bar{X}\right)}^{2}}\;\;$	(3)
			Max SD (ROI)=Mean(ROI)+SD (ROI)	(4)
	Histogram	It represents the distribution of density values of ROI by graphical bars. The sum of these values was calculated using Equation (5). Normalized of histogram values by resetting the density value were calculated by Equation (7).	$sum\;his\;ROI=\sum_{i=1}^{n}H_{i}$ where n refers to bars number and $H_{i}$ refers to each bar value	(5)
			$area\;ROI=N\times P^{2}$ N represents the number of pixels in ROI, and P is the pixel size.	(6)
			$NHist=\frac{sum\;his\;ROI}{area\;ROI}$	(7)
Shape	ROI Compactness	One of the shape circularity measurement techniques in digital images associated with the shape perimeter and its area [53,54,55]. It is calculated by Equation (8) [55].	$Compctness=\frac{Perimeter^{2}}{4\times 3.14\times N}$ N represents the number of pixels.	(8)
	Kurtosis	Kurtosis measures the tailed-level compared to a normal distribution that indicates whether the data looks flat or not [56,57]. It is calculated by Equation (9).	$Kurtosis=\frac{E{\left(x-\bar{x}\right)}^{4}}{s^{4}}$ $\bar{x}$ is the mean of x and s is SD of ROI.	(9)
Texture	Solidity	It illustrates the ratio of the pixels on the convex body in the ROI area by Equation (10) [58].	$Solidity=\frac{Area}{ConvexArea}$	(10)
	Correlation	It measures the correlative value between the pairs of pixels [48,59]. If the gray level between the pixel pair is stable in ROI, it indicates a high correlation value [48]. Equation (11) is used to calculate this feature.	Correlation= $\sum_{i=1}^{n}\sum_{j=1}^{n}\frac{\left(i-\bar{x}\right)\left(j-\bar{x}\right)p\left[i,j\right]}{\sigma^{2}}$ where $\bar{x}$ is the mean and $\sigma^{2}$ is the SD	(11)
	Variance	It is a squared of SD. It is used to measure the degree of dispersion and the variation in the pixels value in the ROI [52,60].	$Variance=S^{2}$	(12)
	Uniformity	In this feature, we make the density in the ROI’s histogram uniform by squaring the normalized histogram value.	$Uniformity=NHist^{2}$	(13)

### 5.4. Experiments and Results

Several experiments on the classifiers mentioned above have improved the models’ performance based on the input dataset. The experiments started with the default models’ properties tuned by functions developed in MATLAB with all the extracted features. These experiments continued until we optimized all the models’ properties and achieved the highest accuracy by integrating one of the features selection functions, which will be explain in detail in the following sub-sections.

#### 5.4.1. First Experiment

The mentioned classification techniques with default properties mode were tested with all features for the model’s training. Two cross-validation (CV) methods were used for the dataset partitioning: Holdout and K-Fold.

**Figure 6:**
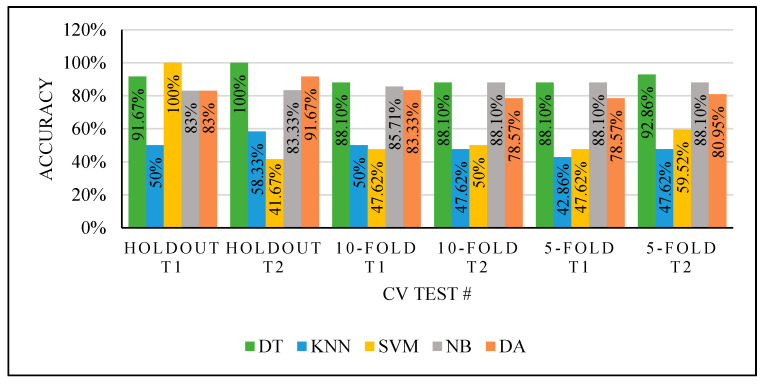
Experiment 1 results.

This experiment obtained different results for each classifier, as seen in Figure 6, with the two different CV partitioning methods creating different training sets. These variations are due to different factors depending on the used classifier. However, the used classifiers with default properties and random selection of the training sets using either Holdout or K-Fold are inefficient. We could not trust the results even if we obtained high performance, which needs improvements.

#### 5.4.2. Second Experiment

Various range criteria were used for manual dataset partitioning with all the extracted features to train and test the classifiers, where each set has a balanced number of benign and malignant cases to improve the models’ performance. The dataset partitioning range categorized the following tests. Only KNN parameters were modified in this experiment because it produced poor performance in all the Experiment 1 tests. The KNN parameters were modified after testing all the distances with 1 to 10 K values with two different partitioning ranges, 70–30% and 75–25%. 

Based on these tests, the best parameters’ values were tuned, as shown in Table 2. Euclidean distance performed very well with different values of K. The selected K value was decided by considering that the small values are more flexible for a fit, but it will increase the variation. The large values use high computational power; thus, the selected value was not very small but also not the largest tested value. The number of neighbors in Table 2 was selected to avoid those possibilities.

**Figure 7:**
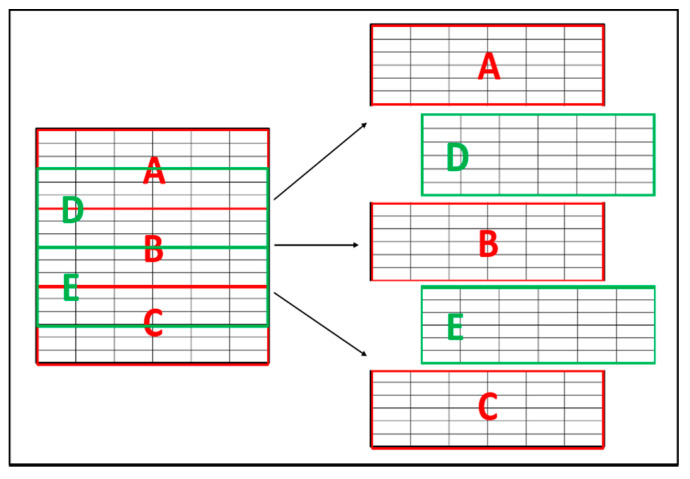
Testing set selection.

In this experiment, we tried to improve all testing classifiers by guaranteeing that the sets after the partitioning process contains balanced data. Four partitioning ranges were used; each range was tested five times, wherein each test, we used a different part from the dataset as a testing set. Figure 7 shows how the split process for the testing set was done.

**Figure 8:**
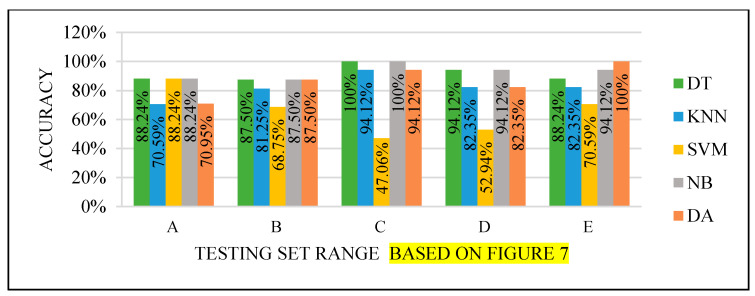
Models’ performance with the dataset splitting criteria at 60% training and 40% testing.

**Figure 9:**
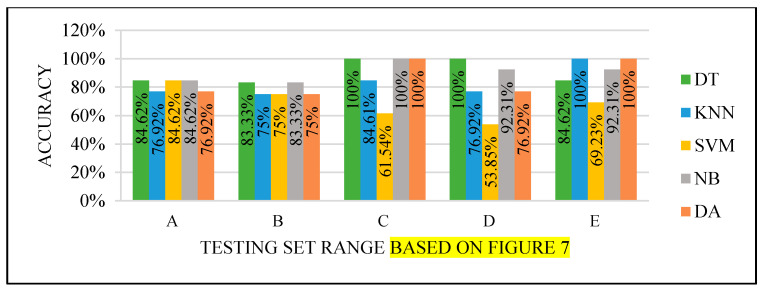
Models’ performance with the dataset splitting criteria at 70% training and 30% testing.

**Figure 10:**
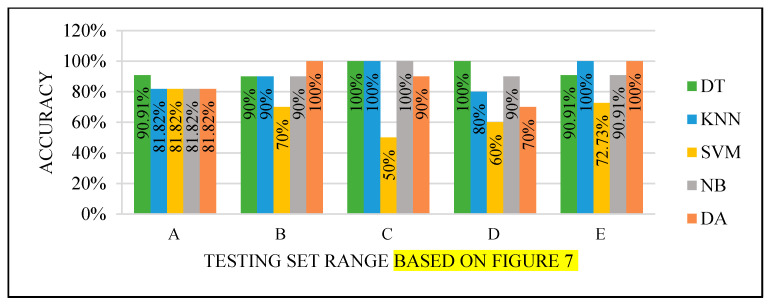
Models’ performance with the dataset splitting criteria at 75% training and 25% testing.

**Figure 11:**
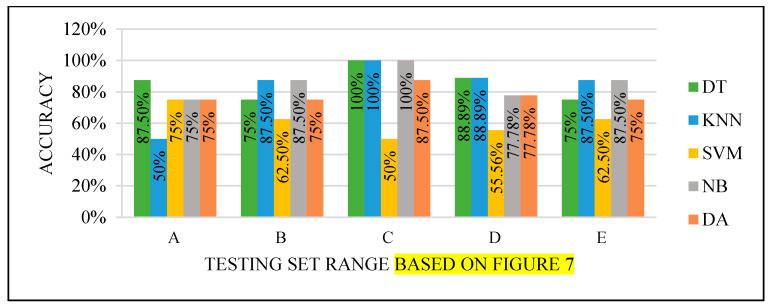
Models’ performance with the dataset splitting criteria at 80% training and 20% testing.

All the test results produced the best performance when using the end of the dataset (C) as the testing set, as seen in Figure 8, Figure 9, Figure 10 and Figure 11. This finding is due to the variety of cases in the training set. We did not manage or control the dataset arrangement, so if the cases used to train the model are similar, it will fail to diagnose other different cases. It means the cases from the beginning of the dataset vary, making the classification process more efficient and accurate. So, the used model was trained with enough variation in the cases to classify the new cases accurately. 

SVM did not perform well, and this could be due to two factors: model properties or data dimensionality. So, it needed to be improved to achieve the highest accuracy efficiently.

All the models needed to be improved to guarantee the results that the end-user can trust with the least computation time and least resource consumption. For this purpose, we integrated two optimization functions in the third experiment that could achieve the best performance, which are explained in the following.

##### Forward Sequential Feature Selection (FSFS)

Its name refers to the repeated nature of the process [61] until obtaining the best feature to achieve accurate classification results. It has a faster computational time where it reduces the needed calculation process of a large number of attributes and saves the computing resources such as memory [61,62].

##### Hyperparameters Optimization Function

Most of the classification techniques’ performance depends on the settings of the hyper-parameters of the classifiers [63,64]. 

In recent studies, Bayesian optimization proved that it performs better than other methods [63,64]. It works based on an iterative Gaussian process, a robust technique for ML [65].

#### 5.4.3. Third Experiment

We combined two functions to improve the classifiers’ accuracy and computational time to obtain an efficient result. The FSFS and hyper-parameters optimization functions were used together.

Cases were selected for the training and testing partitions randomly and manually, resulting in 70% of the dataset for training and 30% for testing. The random process was repeated three times to test the performance with as many possible differences of the selected cases.

##### Random Selection

The dataset was reordered randomly using a function in MATLAB. Then it was partitioned to 70% of the dataset for training and the rest for testing. We repeated this process three times. Every time the test created training and testing sets that contained different cases due to reordering them using the random function. Thus, the aim of the many tests was to experiment with the performance with different sets that contain randomly selected cases. For each created training and testing set, the models were trained using a 10-fold CV, and then tested using a hyper-parameter function to optimize the parameters with all classifiers.

The steps mentioned above were used with the FSFS function to select the best feature (BF) and the best two features (B2F) again.

**Figure 12:**
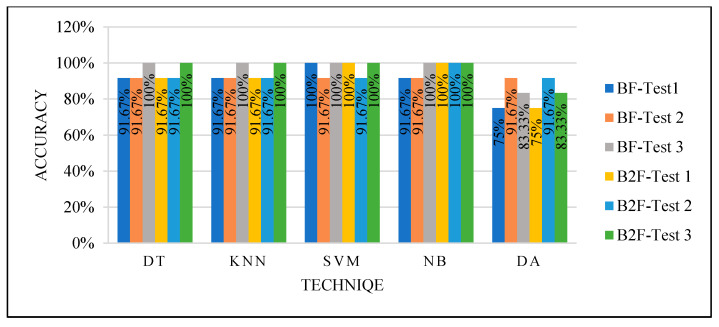
Models’ performance with a random selection of the training set.

We achieved high performance by using randomly selected cases to split the dataset for the training and testing phase and using parameter optimization and feature selection functions that reduced the data dimensionality, as seen in Figure 12. This means the models that needed to be trained with balanced data to perform well, such as DT, can perform very well with unbalanced data once using parameters optimization and dimensionality reduction. However, while we could not control the selected cases variation for training, the results were not efficient enough, and we could not guarantee the performance of the classifiers with new cases.

##### Manual Selection

The dataset manually selected 70% of cases for training and 30% for testing to guarantee a balance and variation in the training cases. The training and testing sets were used to test all the classifiers with the FSFS and parameter optimization functions.

**Figure 13:**
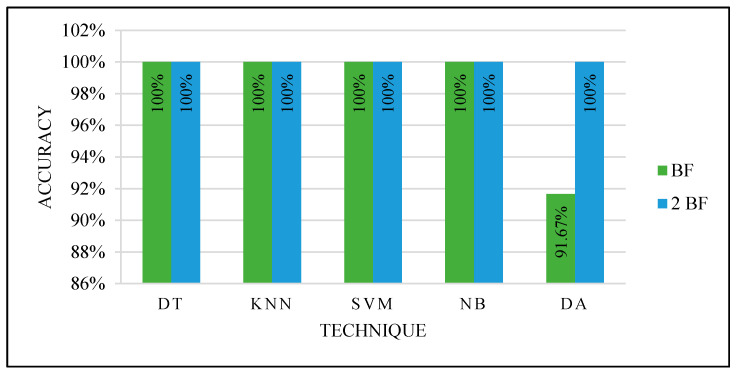
Models’ performance with manual selection of the training set.

They were tested two times: by selecting the best feature and the best two features. Figure 13 shows the classifiers’ performance with both the best and best two features. All the classifiers produced an excellent performance.

We achieved the highest accuracy using manually selected cases to split the dataset for the training and testing phase, the parameter optimization function, and the feature selection function that reduced the data dimensions. This test considered all the factors that may affect the classifiers’ performance. The selected cases were balanced and varied. All the classifiers were modified to produce the optimized parameters, and the dimensions were reduced.

**Figure 14:**
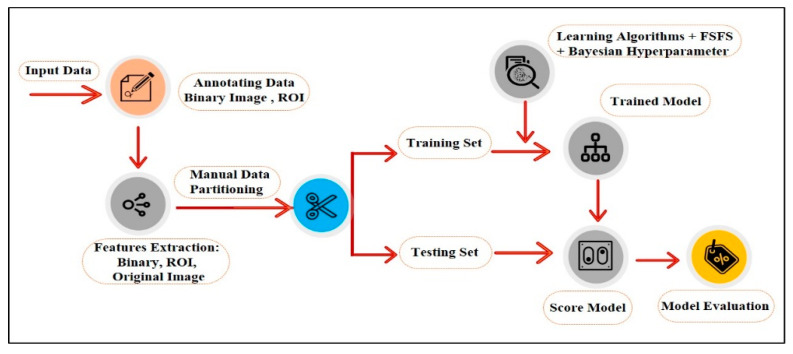
Optimized method.

Based on all the previous experiments where the manual selection of the cases for the training set achieved excellent performance with the best feature and the best two features selection, the proposed method in the previous section shown in Figure 2 was optimized, as shown in Figure 14.

We considered the computational time an essential factor for the end-user when they want to predict a new case using one of the optimized techniques. Predicting one or two extracted features from one image instead of 12 extracted features from three images (original image, ROI, and binary) of one instance will save the needed computation time and computer resources. So, the least features were tested. Once the test performed very well, it means the training and testing sets with the best feature for all the classifiers, except DA, which has an excellent performance with two features, are the most efficient, and meet our expectations. Based on the results, the most crucial feature was the ROI compactness. In general, the shape features gave an excellent performance compared to the other features.

#### 5.4.4. Proving Test

**Figure 15:**
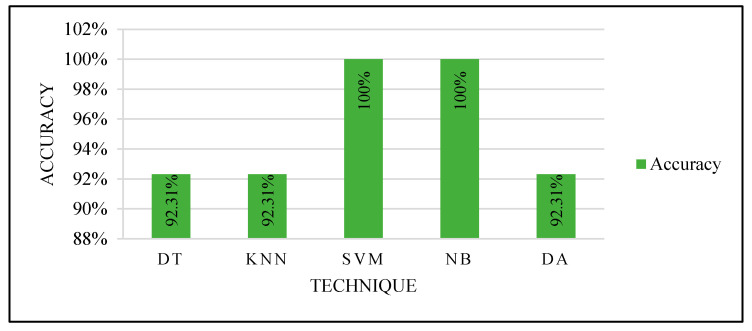
Proving the test results.

To prove the optimized models’ performance with the best features, 13 unlabeled mammograms were collected from KFUH. The collected cases were processed to extract the features and tested with each optimized classifier as in the third experiment with manual partitioning. After the prediction with the optimized models, the results were shown to the radiologist at KFUH to check the correctness of the optimized models. Figure 15 shows the results.

Based on the proving test results, SVM and NB are the most powerful, efficient, and accurate ML techniques. SVM and NB proved their efficiency to solve this problem. Although other techniques performed very well, SVM and NB are the most accurate techniques. In addition, they were able to accurately predict using only one feature: the ROI compactness. This indicates the ability to deal with only one feature that could save the computation time to calculate several features for every input case.

## 6. Discussion

As explained in the previous section, after extracting 12 features from the annotated mammography dataset, several experiments were done with various criteria of partitioning and hyper-parameter optimizations to obtain the highest performance with all the tested classifiers. We compared to determine the best techniques to trust its results. Four factors may affect the performance of the classifiers: data balance, training data variety, data dimensionality, and the used classifiers’ parameters. These factors depend on each classifier.

In the first experiment, we used two CV techniques and tested them several times with all classifiers. The results produced while using the classifiers for each test in this experiment or when using two tests for each classifier indicated that the classifiers’ performances were not stable. That led us to do more experiments with a managed partitioning process to improve the performance of the classifiers.

The second experiment was done by managing the data partitioning process based on the first experiment’s results. Four ranges of dataset splitting were assessed; each range was tested five times where each time we picked the testing set from a different place of the dataset. This experiment showed good performance for DT, NB, and DA. SVM, which is one of the most powerful techniques, was not performing well. So, we planned more experiments to improve its performance and improve the computation time and resources consumption of all the classifiers.

We combined Forward Sequential Feature Selection and Bayesian hyper-parameter optimization functions to improve the results and guarantee the performance efficiency of the tested classifiers. These functions were used with random and manual training and testing sets partitioning. Both were tested by selecting the best feature and the best two features. The test with only one and two features was selected due to needing the least computation time and least resources consumption. This combination gave an excellent performance with 100% accuracy with manual partition because of the various cases selection for the training set, which is an essential factor. 

**Figure 16:**
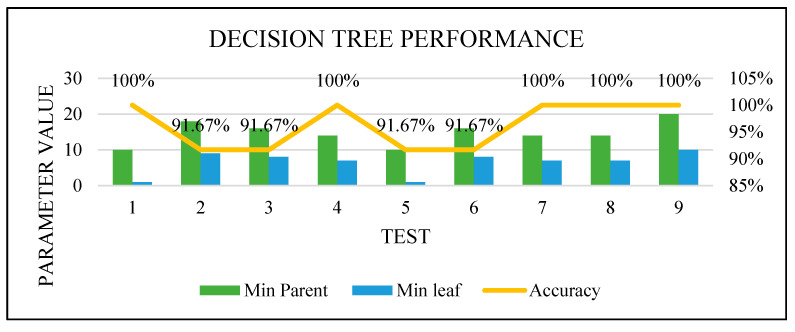
DT optimization.

**Figure 17:**
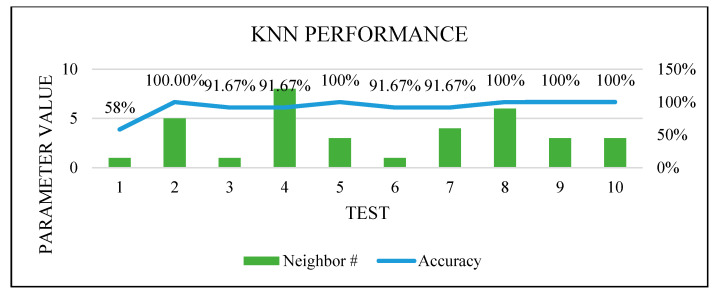
KNN optimization.

**Figure 18:**
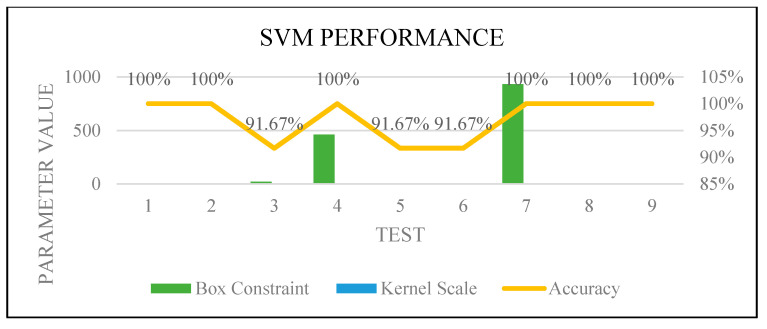
SVM optimization.

**Figure 19:**
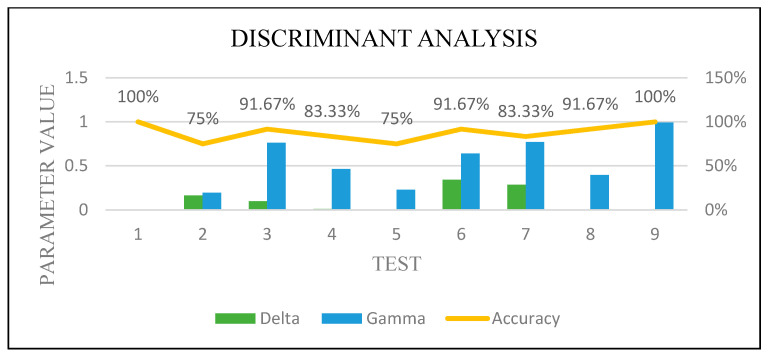
DA optimization.

Figure 16, Figure 17, Figure 18 and Figure 19 show how the performance of each technique was changed among all the experiments. The parameters of NB were not changed, and it worked very well once it was combined with the feature selection function.

The newly trained models with the best features, manual partition, and parameters optimization were used in proving the test using unlabeled data, to be confirmed by the radiologist that the optimized models obtained the aimed performance. 

**Figure 20:**
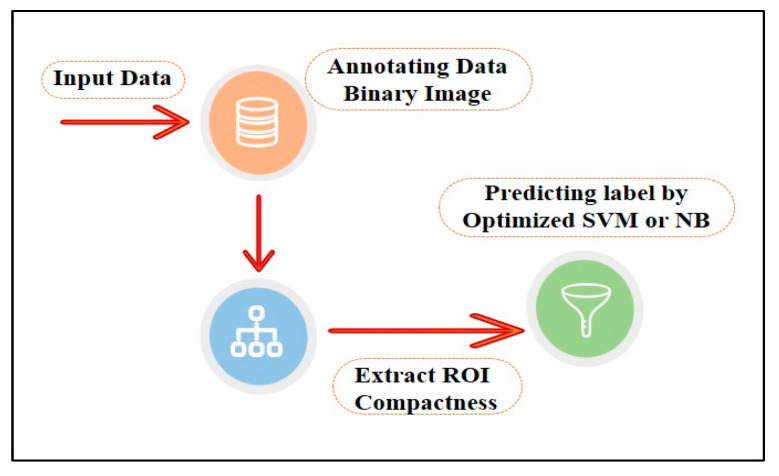
Recommended model.

Based on all experiments findings, the proposed ML-based CAD system is shown in Figure 20. It achieved the highest accuracy with the least computation time and the least resources consumption for input processing. According to the proven test results shown in Figure 15, the optimized models used in the third experiment by selecting the best feature and manually partitioning the dataset led to using the optimized parameters for SVM and NB.

The proposed method for a CAD system can be performed using the following steps. The mammogram needs to be annotated and saved as a binary image of the ROI manually. Then, one should calculate the ROI compactness using Equation (8) in Table 1, where it should be enough, as we proved in the third experiment. Then, based on the proven test results, we recommend performing the classification process by either SVM or NB with its optimized parameters, which resulted in 100% accuracy for the classification process of new cases.

## 7. Conclusions and Future Work

In this study, we developed a breast cancer diagnosis based on an ML method. This method is considered to avoid the possible errors in tumor segmentation by current CAD systems. Errors may occur due to the complex nature of breast tissue. The proposed method uses the radiologist’s knowledge to draw the tumor border using a touchpad. The radiologist’s rule in the proposed method makes the process more accurate and efficient by considering the border smoothness, tailedness, or coarseness. After annotating the ROI, we saved the manually annotated area as a binary image (black and white) and masked image, which presented the tumor only (without its background by showing its margins in black). This was used to extract the ROI features related to density, shape, and texture. Then, we built the diagnostic model to work while considering the least computation time and saving the computer resources.

This study concludes that SVM and NB with tuned parameters are the most powerful and efficient classification techniques based on the ROI compactness, which we extracted using the binary image of ROI, thus achieving the maximum efficiency and resource conservation. We recommend doing more tests with a large number of samples for training the recommended model. In addition, further testing for the other classifiers, either hybrid or individual, is recommended.

## Figures and Tables

**Table 2:** Modified KNN parameters.

Properties	Value
Number of neighbors	5
Distance	Euclidean

## Data Availability

The data presented in this study are available on request from the corresponding author. The data are not publicly available since it was obtained after the approval from Institutional Review Board as mentioned above.
